# *Chlamydia trachomatis* L2c Infection in a Porcine Model Produced Urogenital Pathology and Failed to Induce Protective Immune Responses Against Re-Infection

**DOI:** 10.3389/fimmu.2020.555305

**Published:** 2020-10-26

**Authors:** Evelien De Clercq, Matthias Van Gils, Katelijn Schautteet, Bert Devriendt, Celien Kiekens, Koen Chiers, Wim Van Den Broeck, Eric Cox, Deborah Dean, Daisy Vanrompay

**Affiliations:** ^1^ Laboratory for Immunology and Animal Biotechnology, Department of Animal Production, Faculty of Bioscience Engineering, Ghent University, Ghent, Belgium; ^2^ Laboratory of Immunology, Faculty of Veterinary Medicine, Ghent University, Merelbeke, Belgium; ^3^ Department of Pathology, Bacteriology and Poultry Diseases, Faculty of Veterinary Medicine, Ghent University, Merelbeke, Belgium; ^4^ Department of Morphology, Faculty of Veterinary Medicine, Ghent University, Merelbeke, Belgium; ^5^ Center for Immunobiology and Vaccine Development, Children’s Hospital Oakland, Research Institute, Oakland, CA, United States; ^6^ Department of Medicine, University of California, San Francisco, CA, United States; ^7^ Joint Graduate Program in Bioengineering, University of California, Berkeley, CA, United States

**Keywords:** *****Chlamydia trachomatis*, genital infection, immunology, large animal model, lymphogranuloma venereum, re-infection

## Abstract

The current study was designed to evaluate the pathogenesis, pathology and immune response of female genital tract infection with *Chlamydia trachomatis* L2c, the most recently discovered lymphogranuloma venereum strain, using a porcine model of sexually transmitted infections. Pigs were mock infected, infected once or infected and re-infected intravaginally, and samples were obtained for chlamydial culture, gross and microscopic pathology, and humoral and cell-mediated immunity. Intravaginal inoculation of pigs with this bacterium resulted in an infection that was confined to the urogenital tract, where inflammation and pathology were caused that resembled what is seen in human infection. Re-infection resulted in more severe gross pathology than primary infection, and chlamydial colonization of the urogenital tract was similar for primary infected and re-infected pigs. This indicates that primary infection failed to induce protective immune responses against re-infection. Indeed, the proliferative responses of mononuclear cells from blood and lymphoid tissues to *C. trachomatis* strain L2c were never statistically different among groups, suggesting that *C. trachomatis*-specific lymphocytes were not generated following infection or re-infection. Nevertheless, anti-chlamydial antibodies were elicited in sera and vaginal secretions after primary infection and re-infection, clearly resulting in a secondary systemic and mucosal antibody response. While primary infection did not protect against reinfection, the porcine model is relevant for evaluating immune and pathogenic responses for emerging and known *C. trachomatis* strains to advance drug and/or vaccine development in humans.

## Introduction

*Chlamydia trachomatis* is an obligate intracellular bacterial pathogen that infects annually over 100 million individuals (1). *C. trachomatis* comprises two biovars: the trachoma biovar which includes ocular and urogenital strains and the lymphogranuloma venereum (LGV) biovar (2, 3). The two biovars are serologically subdivided into serovars based on the major outer membrane protein (MOMP) (4). The ocular serovars A, B, Ba and C are primarily associated with trachoma, the leading cause of preventable blindness in developing countries (5). The urogenital (D-K, Da, Ia, and Ja) and LGV (L1-L3, L2a, L2b, and L2c) serovars cause sexually transmitted infections. Urogenital infection with serovars D to K, including Da, Ia, and Ja, can result in cervicitis, urethritis and post-infection complications such as pelvic inflammatory disease, ectopic pregnancy, infertility, chronic pelvic pain, epididymitis and infant pneumonia. The LGV serovars cause a more invasive disease called lymphogranuloma venereum. After transiently infecting epithelial cells, these serovars penetrate into the submucosal tissues to infect macrophages and monocytes and consequently spread to regional draining lymph nodes (6). The disease usually manifests as acute inguinal lymphadenitis with abscess formation (inguinal syndrome) following urogenital inoculation, whereas anorectal entrance of the bacteria can lead to acute hemorrhagic proctitis (anorectal syndrome) (7, 8). Without treatment, persistent infections with chronic inflammation arise, resulting in strictures and fistulas of the involved region, which can eventually result in serious complications such as genital elephantiasis, esthiomene and the frozen pelvis syndrome with infertility (9, 10).

LGV is endemic in parts of Africa, South-East Asia, South America and the Caribbean, and has been considered a rare disease in developed countries until recently (8, 11, 12). Since 2003, LGV outbreaks among men who have sex with men (MSM) have been reported in Europe (13–19), North America (20, 21) and Australia (22). Almost all infected men suffered from severe proctitis, characterized by anorectal pain, haemopurulent discharge and rectal bleeding, whereas genital and inguinal symptoms were rare. A high proportion of LGV patients was also infected with HIV (23). The vast majority of infections was caused by serovar L2b, which was first identified in patients from Amsterdam (24). Recently, a new LGV serovar, called L2c, was isolated from an HIV negative MSM with severe hemorrhagic proctitis. This hypervirulent serovar appeared to be a recombinant of *C. trachomatis* serovars L2 and D (25). The extent of dissemination of serovar L2c or other LGV recombinants within the MSM community still have to be investigated (26). In industrialized countries, LGV is very uncommon in women, although a few asymptomatic female patients or those with cervicitis have been described (27, 28). Recently, the first case of *C. trachomatis* L2b proctitis in a woman was reported (29). Furthermore, Verweij et al. (30) described the first urogenital L2b infection in a female patient with bubonic LGV. Considering the ongoing outbreaks, LGV infections among bisexual and heterosexual men as well as heterosexual women are likely to increase in the near future (30).

Given the importance of LGV infections globally, the purpose of the current study was to investigate the pathogenesis, pathology and immune response of vaginal *C. trachomatis* L2c infection in a relevant animal model. Previously, Vanrompay et al. (31) demonstrated that pigs are a suitable animal model to study female genital tract infection with *C. trachomatis* serovar E strains. Pigs are immunologically, genetically and physiologically more closely related to humans than rodents, and are ethically and practically more convenient than primates.

## Materials and Methods

### *Chlamydia trachomatis* Strain

*C. trachomatis* strain L2c was isolated from the rectal mucosa of a male who had a history of sex with men and suffered from severe hemorrhagic proctitis (25). Bacteria were propagated in McCoy cells using standard procedures (32). The tissue culture infective dose (TCID_50_) of the *C. trachomatis* stock was determined by the method of Spearman and Kaerber (33).

### Animals

Fifteen 9-week-old conventionally bred female pigs (Belgian Landrace) were randomly assigned to three groups of five pigs, each housed in separate isolation units. The animals were fed *ad libitum* with a commercial starting diet. The pigs were seronegative for *Chlamydiaceae* as determined by a *C. suis* ELISA (34). Nasal, rectal and vaginal swabs did not contain chlamydial bacteria as determined by culture on McCoy cells (34).

### Experimental Infection and Euthanasia

On day 0, when pigs were 9 weeks old, all groups were anesthetized by intramuscular injection of Zoletil^®^ 100 (Virbac Animal Health, Louvain La Neuve, Belgium) in 2% Xylazine-M^®^ (VMD, Arendonk, Belgium). The control group and the infected group were inoculated intravaginally with phosphate-buffered saline (PBS). The re-infected group was infected by intravaginal injection of 1 x 10^7^ TCID_50_ of *C. trachomatis* strain L2c. Intravaginal inoculation was performed by inserting an artificial insemination pipette connected to a syringe into the vagina and injecting 1 ml PBS or 1ml of the bacterial suspension in PBS. On day 56, the 17-week-old pigs were anesthetized again. Subsequently, the control group was inoculated with PBS, whereas the infected group and the re-infected group were infected intravaginally with *C. trachomatis* strain L2c (1 x 10^7^ TCID_50_). At day 77, when pigs were 21 weeks old, all animals were euthanized by intravenous injection of an overdose of pentobarbital (70 mg/kg; Nembutal^®^, Ceva Santé Animale, Maassluis, the Netherlands) followed by exsanguination. All animal procedures were in accordance with the guidelines of the animal care and ethical committee of Ghent University.

### Sample Collection and Processing

Vaginal swab samples in 2 ml sucrose-phosphate transport medium (2-SP) for chlamydial isolation as well as in 2 ml PBS with protease inhibitor for mucosal antibody detection were collected weekly. These samples were stored at -80°C until tested. Additionally, blood samples (*v. jugularis*) for antibody detection were taken weekly. Blood was stored overnight at room temperature and centrifuged (9300 × *g*, 20°C, 10 min) to collect serum. Sera were stored at -20°C until analysis. Peripheral blood mononuclear cells (PBMC) were isolated at 7 and 10 days post infection or re-infection to determine their proliferative responses and immune cell subpopulations.

At euthanasia, the entire body of the pigs was examined for gross lesions that were scored as none (0), slight (1), moderate (2) or severe (3). Mononuclear cells (MC) from the spleen, the cervical lymph node (*lymphonodulus cervicalis superficialis)* and the pelvic lymph nodes (the *lymphonoduli iliaci mediales*, the *lymphonoduli iliaci laterales*, the *lymphonoduli sacrales* and the *lymphonoduli anorectales*) were collected to analyze the proliferative responses and immune cell subpopulations. Samples of the spleen, liver, pelvic lymph nodes, caecum, urethra, vagina, cervix, corpus uteri, uterine tubes, oviducts and ovaries were imbedded in methylcellulose medium, frozen in liquid nitrogen and stored at -80°C until preparation of cryostat tissue sections for the detection of *C. trachomatis* antigen. Samples from the same tissues were fixed in 10% phosphate-buffered formalin for histopathology.

### *C. trachomatis* Detection in Swabs and Tissue Sections

Vaginal swabs in 2-SP were shaken for 1 h at 4°C, centrifuged (1200 x g, 1 h, 37°C) and 75 µl of the swab content was inoculated on McCoy cells grown on 13 mm cover slips in *Chlamydia* Trac Bottles (International Medical, Brussels, Belgium) using standard techniques (32). Before the inoculum was added to the cells, they were washed twice with 1 ml phosphate buffer supplemented with 0.003% DEAE-dextran. Chlamydial growth was analyzed using the Mikrotrak direct immunofluorescence staining (Kordia, Leiden, The Netherlands) at 6 days post inoculation. Cryostat tissue sections (5 µm) were also stained by use of the Mikrotrak immunofluorescence test. All slides were examined by immunofluorescence microscopy (BX41 Olympus, 600×). *C. trachomatis* positive cells were counted in five randomly selected microscopic fields. A score ranging from 0 to 6 was given for each swab or tissue section. Score 0 indicated that there were no *C. trachomatis* inclusion forming units (IFU). Score 1 and 2 indicated a mean of 1 to 5 and 6 to 10 small IFU per microscopic field, respectively. Score 3 represented more than 10 small IFU and 1 larger IFU per microscopic field. Score 4, 5, and 6 indicated 1 to 5, 6, to 10 and more than 10 larger IFU per microscopic field, respectively.

### Histopathology

Tissue samples were fixed in 10% phosphate-buffered formalin, dehydrated and embedded in paraffin, sectioned at 5 µm and stained with hematoxylin and eosin. Slides were examined blindly by a veterinary pathologist. The microscopic findings were either graded (none (0), minimal (1), slight (2), moderate (3), marked (4) or severe (5) histological change) or indicated as present or absent without a grade.

### Serum and Mucosal Antibody Analysis

Sera were heat inactivated at 56°C during 30 min and subsequently pretreated with kaolin to reduce background signals in ELISA (35). Vaginal swabs in PBS with protease inhibitor were shaken for 1 h at room temperature. Isotype-specific serum and mucosal antibody titers were determined using a *C. trachomatis* L2c ELISA. Briefly, Maxisorp 96-well microtiter plates were coated with purified *C. trachomatis* strain L2c EBs (1 × 10^7^ TCID_50_ per well) diluted in PBS. Plates were blocked overnight with PBS supplemented with 5% bovine serum albumin (BSA) at 4°C. Antibody titers were determined using twofold dilution series in dilution buffer (PBS + 3% BSA + 0.05% Tween^®^20), starting at a dilution of 1:15. Serum and swab samples from a previous experimental infection in pigs (36) were used as positive and negative controls. Antibody isotype titers were analyzed using monoclonal antibodies against swine IgA (mAb 27.8.1), IgG (mAb 23.3.1b), and IgM (mAb 28.4.1) (37) at a dilution of 1:15, 1:20, and 1:50, respectively, followed by an 1:5,000 dilution of biotinylated anti-mouse IgG (H+L) (Dako, Glostrup, Denmark). Subsequently, the plates were incubated with 1:2500 diluted peroxidase-labeled streptavidin (Zymed Laboratories, San Francisco, USA). Finally, the substrate and chromogene ABTS (2, 2’-Azino-di(3-ethylbenzthiazoline-6-sulfonate); KPL, Maryland, USA) was added. Antibody titers were defined as the inverse of the highest sample dilution giving an absorbance value at 405 nm above the cut-off value (mean absorbance of seronegative pig serum at a dilution of 1:15 + twice the standard deviation).

### PBMC and MC Proliferation Assay

At 7 and 10 days post infection or re-infection, PBMC were isolated from heparinized blood samples collected from the jugular vein by density gradient centrifugation (500 × g, 18°C, 25 min) on Lymphoprep™ (Axis-Shield, Oslo, Norway). Subsequently, the erythrocytes were lysed with ammonium chloride. After centrifugation (270 × g, 4°C, 10 min), the cells were washed and resuspended in leukocyte medium (RPMI-1640 (Life Technologies, Merelbeke, Belgium) supplemented with 5% heat-inactivated fetal calf serum (Life Technologies), 5×10^−5^ M β-mercaptoethanol (Life Technologies), 1% non-essential amino acids (Life Technologies), 1% sodium pyruvate (Life Technologies), 1% L-glutamine (Life Technologies), 1% penicillin-streptomycin and 1% kanamycin).

At euthanasia, mononuclear cells (MC) were isolated from the spleen, the cervical lymph node (*cervicalis superficialis)* and the pelvic lymph nodes (*iliaci mediales*, *iliaci laterales, sacrales* and *anorectales*). Isolation of MC was performed by mincing the tissues, after removal of the surrounding fat. Erythrocytes were lysed with NH_4_Cl solution and the cells were washed and subsequently resuspended in leukocyte medium without β-mercaptoethanol. PBMC or MC were placed in 96-well tissue culture plates at a concentration of 5 × 10^5^ cells per well. Proliferative responses were tested by adding 10^5^ C*. trachomatis* purified L2c EBs, 10 µg concanavalin A (ConA) (positive control) or medium (negative control) to the wells. Each condition was tested in duplicate. The cells were incubated at 37°C in a humidified atmosphere with 5% CO_2_. ConA- or antigen-induced proliferation was measured by incorporation of 1 µCi/well of ^3^H-thymidine (Amersham ICN, Bucks, UK) for the last 16 h of a 3-day or 4-day culture period, respectively. Cells were harvested onto glass fiber filter strips (Perkin Elmer, Life Science, Oosterhout, The Netherlands) with a cell harvester (Skatron, Liers, Norway) and radioactivity was measured with a β-scintillation counter (Perkin Elmer). As a measure of proliferative response, stimulation indices (SI) were calculated as the ratio of the mean counts per minute of stimulated PBMC or MC versus the mean counts per minute of the negative control.

### Flow Cytometric Analysis of Immune Cell Subpopulations

The immune cell subpopulations in the blood, the spleen, the cervical and pelvic lymph nodes were examined by flow cytometry. T cell populations (CD3^+^CD4^+^CD8^-^, CD3^+^CD4^-^CD8^+^, CD3^+^CD4^+^CD8^+^, CD3^+^CD4^-^CD8^-^, T cells with a γδ T cell receptor), B cells (MHCII^+^CD21^+^, MHCII^+^IgM^+^), monocytes (MHCII^+^SWC3^+^), NK cells (CD3^-^CD4^-^CD8^+^) and plasmacytoid dendritic cells (pDC) (CD3^-^CD4^+^CD8^-^) were analyzed. For the lymphoid tissues the CD3^-^CD4^+^CD8^-^ population does not exist entirely of pDC, but also contains an unknown lineage, presumably of myeloid origin (38). It was impossible to distinguish between both populations based on the used markers. PBMC and MC (10^6^ cells) were incubated for 20 min at 4°C in staining buffer (RPMI-1640 + 1% heat-inactivated FCS) with optimal concentrations of monoclonal antibodies (**Supplementary Table 1**). Cells stained with isotype-matched irrelevant mAbs were used as a negative control. After incubation, the cells were washed with staining buffer and stained with the appropriate isotype-specific Alexa-647-, FITC-, or PE- conjugated antibodies (Life Technologies) for 20 min at 4°C. Cells were washed twice and resuspended in PBS. Data were acquired on a FACSCanto flow cytometer (Beckton Dickinson, Erembodegem, Belgium) with a minimum event count of 50 000 and analyzed with FACSDiva^®^ software. Doublets were excluded based on FSC-H/FSC-A and SSC-H/SSC-A plots.

### Statistical Analysis

Statistical analyses were performed using SPSS 22. The non-parametric Mann-Whitney U test was used to analyze differences between two groups. Results were considered significantly different if P<0.05.

## Results

### Gross Lesions

**Figure 1:**
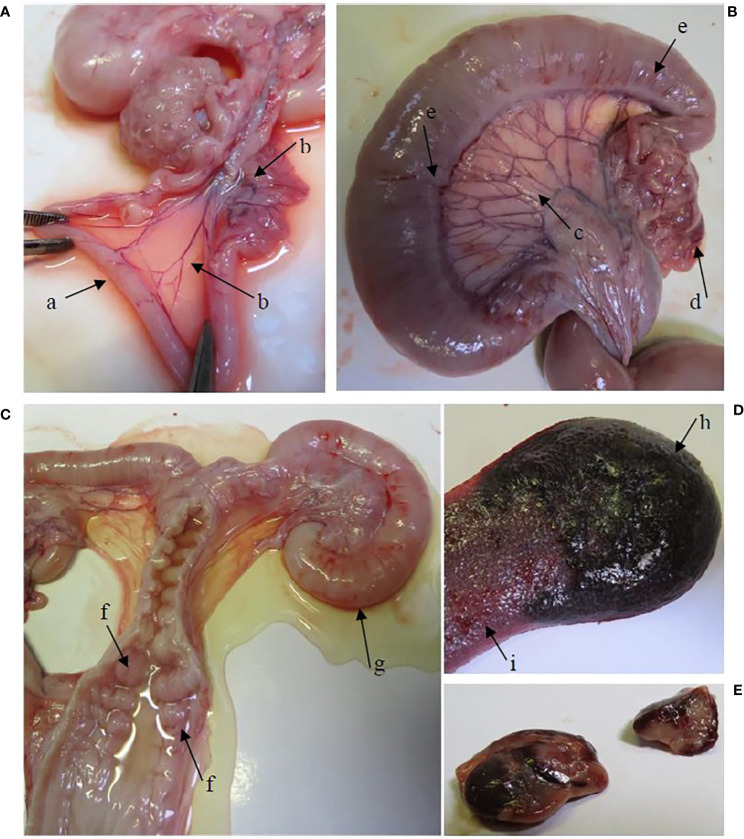
Gross lesions in the re-infected group. **(A)** The oviducts were dilated by the presence of serous exudate in their lumen (arrow a). Hyperemia of the mesovarium and mesosalpinx (arrow b). **(B)** Hyperemia of the *lig. latum uteri* (arrow c), the mesovarium and mesosalpinx (arrow (d) and the uterine tubes (arrow e). **(C)** Hyperemia of the cervical mucosa (arrow f). A large amount of serous exudate was detected in the lumen of the vagina, cervix, corpus uteri and uterine tubes. Note the severely dilated uterine tube (arrow g). **(D)** The spleen is enlarged, hemorrhagic (arrow h) and shows expansion of the red pulp (arrow i). **(E)** Enlarged and hemorrhagic pelvic lymph nodes.

**Supplementary Table 2** shows the median scores for gross lesions in the infection and the re-infected group, determined at necropsy. Gross lesions were absent in all pigs of the control group. Gross pathology was generally more severe for the re-infected group than for the infected group, especially in the urogenital tract. All re-infected pigs showed congestion of the urogenital tract (**Figure 1**), while only three pigs (60%) of the infected group had a congested urogenital tract. A significantly higher congestion of the uterus, uterine tubes, *ligamentum latum uteri*, mesovarium and urethra was noticed in the re-infected group than in the control and/or the infected group. A large amount of clear watery exudate was present in almost the entire genital tract of 40% of the pigs of both infected groups and in the uterus of one animal (20%) of the re-infected group (**Figure 1**). The liver of two re-infected pigs (40%) was moderately congested, whereas the liver was normal in all animals of the infected group. Gross pathology was detected in the spleen of three pigs (60%) of the infected group and four pigs (80%) of the re-infected group (**Figure 1**). The pelvic lymph nodes were moderately to severely enlarged in all animals of both infected groups. Furthermore, moderate to severe congestion of the pelvic lymph nodes was detected in 80% of the pigs of both infected groups (**Figure 1**). Gross lesions in the pelvic lymph nodes were significantly different between the control group and both infected groups.

### Histopathology

**Figure 2:**
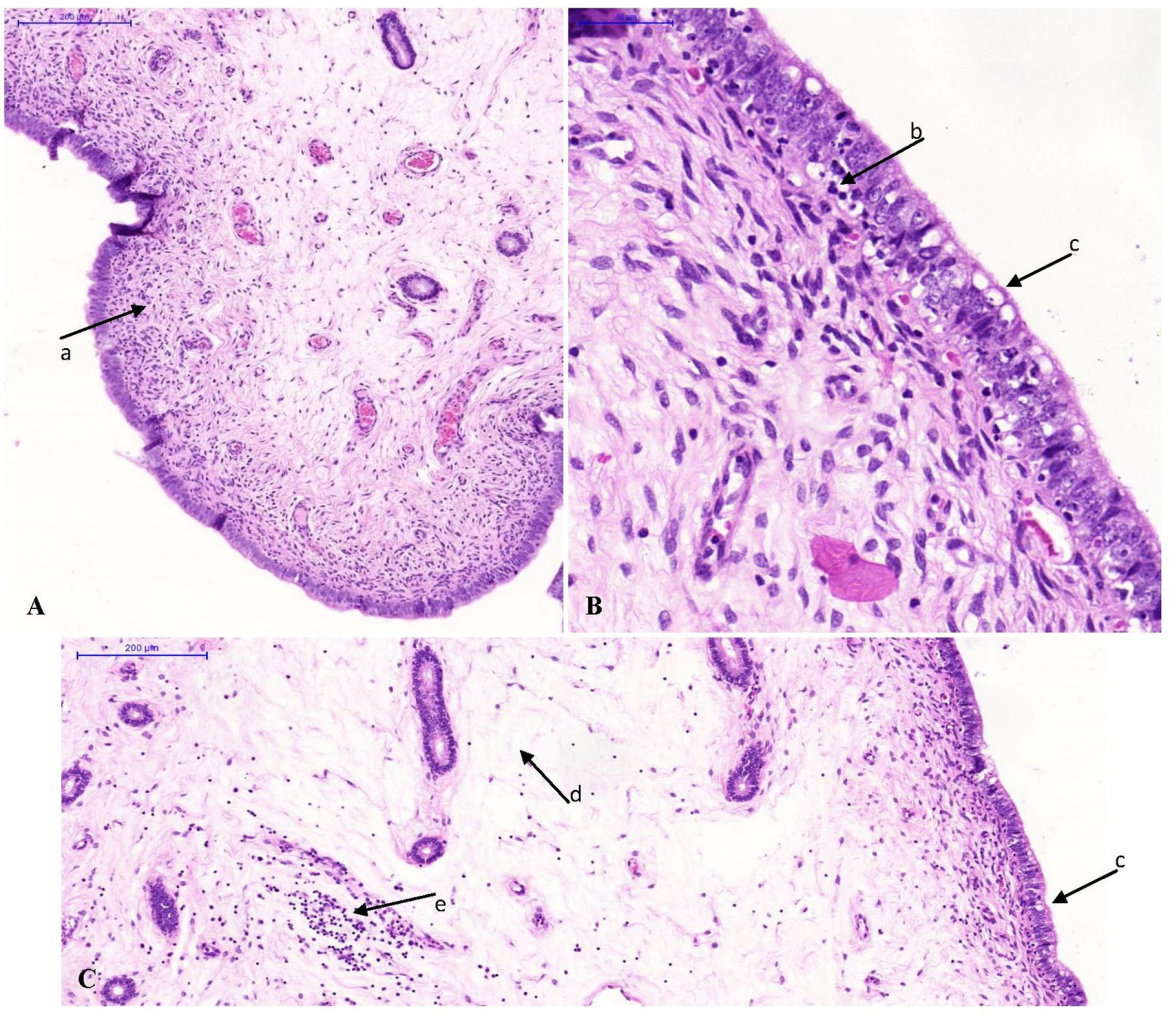
Photomicrographs of uterine horn tissue sections stained with hematoxylin and eosin. Original magnification ×10 **(A, C)** or ×40 **(B)**. **(A)** Minimal lymphocytic infiltration in the lamina propria (arrow a) of a control animal. **(B, C)** Inflammation of the uterine horns of an animal of the infected group. Note the interepithelial lymphocytic infiltration (arrow b), the vacuolation of the epithelium (arrow c), the oedema in the lamina propria (arrow d) and the lymphocytic infiltration in the lamina propria (arrow e).

The median scores of the histopathological lesions detected in the urogenital tract of the pigs are presented in **Supplementary Table 3**. Statistical differences among groups were only present in the cervix, the corpus uteri, the uterine horns and the urethra. In the cervix and the uterine horns, interepithelial inflammatory cells had increased slightly in the mucosa of the infected group (**Figure 2**). Furthermore, epithelial vacuolation was noted in the cervix, corpus uteri and uterine horns of the infected group (**Figure 2**). This vacuolation was to a lesser extent also present when animals were re-infected. The significantly higher median score for epithelial vacuolation in the urethra of the re-infected group was due to the absence of the mucosa in some samples and the high value in one animal. In the oviducts, exfoliated epithelial cells and proteinaceous fluid were observed only in both infected groups, however with low incidence and/or severity. The lesions noticed in the oviducts and the vagina were not statistically different among groups. No histopathological lesions were observed in the ovaries. In the liver of one or two animals of each group minimal to slight focal to multifocal mononuclear inflammations within the parenchyma or portal areas were noted. This lesion is considered to be a background lesion, frequently observed and unrelated to the experimental infection. The liver samples from the other pigs and all spleen, caecum and pelvic lymph node samples showed no microscopic lesions.

### *Chlamydia trachomatis* Vaginal Excretion

Vaginal swabs were examined for the presence of viable *C. trachomatis* using culture in McCoy cells. Median culture scores for vaginal swabs of the infection and the re-infected group, collected at different time points post infection, are presented in **Supplementary Table 4**. All vaginal swab samples of the control group were *Chlamydiaceae* negative, as were the swabs of the other pigs taken before primary infection. The infected group vaginally excreted *C. trachomatis* from day 59, three days after primary infection of this group, onwards. In the re-infected group, vaginal *C. trachomatis* shedding was consistently high from day 3 to day 49 post primary infection and was slightly decreased at day 56 and day 59. Median culture scores were again consistently high from seven days post re-infection (day 63) onwards. From day 3 to day 56, vaginal shedding in the re-infected group was significantly higher than in the control group and the infected group, which was not yet infected at these time points. After primary infection of the infected group, the median excretion scores of the infected group and the re-infected group did not differ statistically from each other, but were significantly higher than those of the control group.

### *C. trachomatis* Detection in Tissue Sections

**Table 1** shows the median scores for the presence of *C. trachomatis* in tissues of the infection and the re-infected group at euthanasia. All tissue samples from the control group were negative. *C. trachomatis* was neither detected in the caecum, the spleen, the liver and the pelvic lymph nodes of both infected groups. Chlamydial EBs and intracellular inclusions, containing replicating chlamydiae, were detected in both the lower and upper genital tract tissues of all pigs of the infected and the re-infected group. Thus, an ascending *C. trachomatis* infection occurred in all pigs of both infected groups. Furthermore, chlamydial replication was observed in the urethra of three pigs (60%) of the infected group and two pigs (40%) of the re-infected group. The urethra of the remaining infected animals contained only elementary bodies, except for the urethra of one pig of the re-infected group, which was negative. For all urogenital tissues, median scores were significantly higher for the infected and the re-infected group than for the control group. Statistical analysis revealed no significant differences between the median scores of both infected groups.

**Table 1:** Presence of *C. trachomatis* in tissues of the infected and the re-infected group at euthanasia^#^.

Tissue	Median score* (range) in:	
	Infected group	Re-infected group
Vagina	3 (3-4)*^a^*	3 (1-3)*^b^*
Cervix	3 (3-4)*^a^*	3 (3-3)*^b^*
Corpus uteri	3 (2-4)*^a^*	3 (3-4)*^b^*
Uterine horn R	3 (3-4)*^a^*	3 (1-4)*^b^*
Uterine horn L	3 (3-4)*^a^*	3 (3-5)*^b^*
Oviduct R	3 (3-3)*^a^*	3 (3-4)*^b^*
Oviduct L	3 (3-4)*^a^*	3 (1-4)*^b^*
Ovary R	3 (3-3)*^a^*	3 (0-4)*^b^*
Ovary L	3 (3-3)*^a^*	3 (1-4)*^b^*
Urethra	3 (1-4)*^a^*	1 (0-3)*^b^*

^#^The median score (range) in the control group was 0 (0-0) for all tissues.

*C. trachomatis positive cells were counted in five randomly selected microscopic fields. Score 0: no C. trachomatis positive cells; Score 1: 1–5 EBs and no inclusions; Score 2: 6–10 EBs and no inclusions; Score 3: >10 EBs and 1 inclusion-positive cell; Score 4: 1–5 inclusion-positive cells; Score 5: 6–10 inclusion-positive cells; Score 6: >10 inclusion-positive cells.

a P < 0.05 for a comparison of the control group and the infected group.

b P < 0.05 for a comparison of the control group and the re-infected group.

c P < 0.05 for a comparison of the infected group and the re-infected group.

### Serum and Mucosal Antibody Responses

**Tables 2** and **3** show the *C. trachomatis* L2c-specific IgM, IgG and IgA antibody titers in sera and vaginal secretions collected at different time points post infection or re-infection. Chlamydial antibodies were never detected in the serum and vaginal swab samples of the control pigs. The pigs of the infected and the re-infected group neither had serum or vaginal *C. trachomatis*-specific antibodies before they were infected. Serum IgM, IgG and IgA antibodies were elicited from 7 days post primary infection onwards and peak titers were reached at 14 or 21 dpi. Anti-chlamydial IgM and IgA were observed in serum samples until 21 and 28 days post primary infection, respectively. Serum IgG antibodies, on the other hand, remained detectable throughout the experiment in most pigs. *C. trachomatis* L2c-specific serum IgM, IgG, and IgA titers increased again following re-infection. Serum IgA titers reached peak levels early after re-infection (7 days), whereas serum IgM and IgG titers continued to increase until the end of the experiment. Vaginal secretions were positive for anti-chlamydial IgM, IgG and IgA from 14 to 21, 14 to 28, and 7 to 21 days post primary infection, respectively. Mucosal antibodies appeared again from 7 (IgM and IgG) or 14 (IgA) days post re-infection onwards. Re-infection clearly resulted in a secondary systemic and mucosal antibody response, with significantly lower IgM titers and significantly higher IgG and IgA titers than following primary infection.

**Table 2:** C*. trachomatis* L2c-specific IgM, IgG, and IgA serum antibody titers of the infected (I) and the re-infected (R) group^#^.

Dpi	Procedure	Median titer (range) for:					
		Serum IgM		Serum IgG		Serum IgA	
		Infected group	Re-infected group	Infected group	Re-infected group	Infected group	Re-infected group
0	Infection R	0 (0-0)	0 (0-0)	0 (0-0)	0 (0-0)	0 (0-0)	0 (0-0)
7		0 (0-0)*^c^*	60 (30-60)*^b^* ^,^ *^c^*	0 (0-0)*^c^*	60 (30-60)*^b^* ^,^ *^c^*	0 (0-0)	0 (0-15)
14		0 (0-0)*^c^*	480 (120-480)*^b^* ^,^ *^c^*	0 (0-0)*^c^*	240 (120-240)*^b^* ^,^ *^c^*	0 (0-0)*^c^*	60 (60-60)*^b^* ^,^ *^c^*
21		0 (0-0)*^c^*	240 (60-240)*^b^* ^,^ *^c^*	0 (0-0)*^c^*	120 (60-120)*^b^* ^,^ *^c^*	0 (0-0)*^c^*	30 (30-60)*^b^* ^,^ *^c^*
28		0 (0-0)	0 (0-0)	0 (0-0)*^c^*	30 (30-60)*^b^* ^,^ *^c^*	0 (0-0)*^c^*	30 (15-30)*^b^* ^,^ *^c^*
35		0 (0-0)	0 (0-0)	0 (0-0)	0 (0-30)	0 (0-0)	0 (0-0)
42		0 (0-0)	0 (0-0)	0 (0-0)*^c^*	30 (0-30)*^b^* ^,^ *^c^*	0 (0-0)	0 (0-0)
49		0 (0-0)	0 (0-0)	0 (0-0)*^c^*	30 (0-60)*^b^* ^,^ *^c^*	0 (0-0)	0 (0-0)
56	Infection I & R	0 (0-0)	0 (0-0)	0 (0-0)*^c^*	30 (0-60)*^b^* ^,^ *^c^*	0 (0-0)	0 (0-0)
63		60 (30-60)*^a^*	30 (30-60)*^b^*	30 (30-60)*^a^* ^,^ *^c^*	120 (60-240)*^b^* ^,^ *^c^*	0 (0-15)*^c^*	120 (60-120)*^b^* ^,^ *^c^*
70		480 (120-480)*^a^* ^,^ *^c^*	60 (30-60)*^b^* ^,^ *^c^*	120 (120-240)*^a^* ^,^ *^c^*	480 (120-480)*^b^* ^,^ *^c^*	15 (15-15)*^a^* ^,^ *^c^*	30 (30-30)*^b^* ^,^ *^c^*
77	Euthanasia	120 (60-120)*^a^*	60 (60-120)*^b^*	240 (240-480)*^a^* ^,^ *^c^*	960 (480-1920)*^b^* ^,^ *^c^*	30 (30-30)*^a^* ^,^ *^c^*	15 (15-15)*^b^* ^,^ *^c^*

^#^The median IgM, IgG, and IgA serum antibody titers (range) in the control group were 0 (0-0) for all time points.

a P < 0.05 for a comparison of the control group and the infected group.

b P < 0.05 for a comparison of the control group and the re-infected group.

c P < 0.05 for a comparison of the infected group and the re-infected group.

**Table 3:** C*. trachomatis* L2c-specific IgM, IgG, and IgA vaginal secretion antibody titers of the infected (I) and the re-infected (R) group^#^.

Dpi	Procedure	Median titer (range) for:					
		Mucosal IgM		Musocal IgG		Mucosal IgA	
		Infected group	Re-infected group	Infectedgroup	Re-infected group	Infected group	Re-infected group
0	Infection R	0 (0-0)	0 (0-0)	0 (0-0)	0 (0-0)	0 (0-0)	0 (0-0)
7		0 (0-0)	0 (0-0)	0 (0-0)	0 (0-0)	0 (0-0)*^c^*	15 (0-30)*^b^* ^,^ *^c^*
14		0 (0-0)*^c^*	60 (30-60)*^b^* ^,^ *^c^*	0 (0-0)*^c^*	60 (30-60)*^b^* ^,^ *^c^*	0 (0-0)*^c^*	15 (0-15)*^b^* ^,^ *^c^*
21		0 (0-0)*^c^*	30 (15-30)*^b^* ^,^ *^c^*	0 (0-0)*^c^*	120 (30-120)*^b^* ^,^ *^c^*	0 (0-0)	0 (0-0)
28		0 (0-0)	0 (0-0)	0 (0-0)*^c^*	15 (0-30)*^b^* ^,^ *^c^*	0 (0-0)	0 (0-0)
35		0 (0-0)	0 (0-0)	0 (0-0)	0 (0-0)	0 (0-0)	0 (0-0)
42		0 (0-0)	0 (0-0)	0 (0-0)	0 (0-0)	0 (0-0)	0 (0-0)
49		0 (0-0)	0 (0-0)	0 (0-0)	0 (0-0)	0 (0-0)	0 (0-0)
56	Infection I & R	0 (0-0)	0 (0-0)	0 (0-0)	0 (0-0)	0 (0-0)	0 (0-0)
63		0 (0-0)*^c^*	30 (30-30)*^b^* ^,^ *^c^*	0 (0-0)*^c^*	60 (30-60)*^b^* ^,^ *^c^*	0 (0-0)	0 (0-0)
70		60 (60-60)*^a^* ^,^ *^c^*	15 (0-30)*^b^* ^,^ *^c^*	60 (30-60)*^a^* ^,^ *^c^*	120 (120-120)*^b^* ^,^ *^c^*	15 (0-15)*^a^* ^,^ *^c^*	30 (30-30)*^b^* ^,^ *^c^*
77	Euthanasia	0 (0-30)	0 (0-0)	120 (60-120)*^a^* ^,^ *^c^*	60 (60-60)*^b^* ^,^ *^c^*	0 (0-15)*^c^*	30 (30-60)*^b^* ^,^ *^c^*

^#^The median IgM, IgG, and IgA mucosal antibody titers (range) in the control group were 0 (0-0) for all time points.

a P < 0.05 for a comparison of the control group and the infected group.

b P < 0.05 for a comparison of the control group and the re-infected group.

c P < 0.05 for a comparison of the infected group and the re-infected group.

### PBMC and MC Proliferation

At 7 and 10 days post primary infection or re-infection, proliferative responses of PBMC to *C. trachomatis* strain L2c were measured. Furthermore, proliferation of spleen, cervical and pelvic lymph node MC was determined at euthanasia. Surprisingly, the proliferative activities of PBMC and MC were never statistically different among groups (data not shown). The proliferative responses to ConA were always positive indicating the viability of the cells and the functionality of the assay.

### Immune Cell Subpopulations

**Figure 3:**
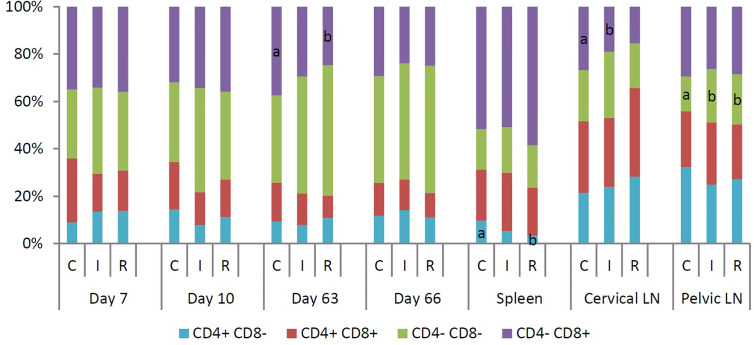
Median percentage of different subpopulations within total T cells (CD3^+^), isolated at 7 and 10 days post infection or re-infection, and in spleen, cervical and pelvic lymph nodes at euthanasia. T cells were divided into four subpopulations based on the expression of CD4 and CD8. For each time point or tissue, different letters within a data series are significantly different (P<0.05). C: control group; I: infected group; R: re-infected group.

Flow cytometry was used to identify blood immune cell subpopulations at 7 and 10 days post primary infection or re-infection (**Figure 3**). Seven days after primary infection of the re-infected group, the median percentage of NK cells was significantly higher for the re-infected group than for the infected group. Three days later, the re-infected group had significantly higher median percentages of T cells, γδ T cells and NK cells than the non-infected animals. At day 63, 7 days after primary infection of the infected group and re-infection of the re-infected group, a significantly higher median percentage of γδ T cells was noticed in both infected groups than in the control group. Within total T cells, significantly fewer CD4^-^CD8^+^ T cells were detected in the re-infected group than in the control group. Moreover, the re-infected animals had significantly lower median percentages of B cells and monocytes than the other groups. At day 66, the re-infected group had significantly lower median percentages of T cells and IgM^+^ B cells, but a significantly higher median percentage of plasmacytoid dendritic cells (pDC) than the other groups. At that time, the median percentages of T cells and γδ T cells were significantly higher for the infected group than for the control group. Furthermore, significantly lower median percentages of mature B cells and monocytes were observed in both infected groups than in the control group. At euthanasia, immune cell populations in the spleen, cervical and pelvic lymph nodes were also determined using flow cytometry (**Figure 3**). For the spleen, significantly higher median percentages of CD4^+^CD8^-^ T cells and CD3^-^CD4^+^CD8^-^ MC, containing pDC, besides a significantly lower median percentage of total T cells were detected in the non-infected animals. The re-infected group had a significantly higher median percentage of NK cells in the spleen than the control group. Furthermore, a significantly lower percentage of monocytes was present in the spleen of the infected group as compared to the other groups. For the cervical lymph nodes, significantly lower median percentages of CD4^-^CD8^+^ T cells and mature B cells were noticed in the infected group than in the control and the re-infected group, respectively. For the pelvic lymph nodes, the re-infected pigs had a significantly lower median percentage of B cells and a significantly higher median percentage of NK cells. Within total T cells, significantly more CD4^-^CD8^-^ T cells were observed in both infected groups than in the control group.

## Discussion

Since 2003, an ongoing outbreak of LGV has been observed among MSM in developed countries (13–22). There have only been a few anecdotal reports of LGV urogenital and rectal infections among women. However, this may change in the near future due to dissemination of the disease to bisexual men and to women (30). While initial infection in the cervix may more likely be asymptomatic compared to rectal infections in males and thus remain untreated, both men and women develop serious complications from LGV infections. Because the porcine model represents an appropriate model for studying human *C. trachomatis* strains (31), the present study aimed to obtain more knowledge about the pathogenesis, pathology and immune response of female genital tract infection and re-infection with *C. trachomatis* L2c, the most recently discovered LGV strain (31).

At necropsy, gross pathology was generally more severe for the re-infected pigs than for animals infected only once. In women, repeated urogenital *C. trachomatis* infections are also associated with an elevated risk of pathological damage resulting in reproductive complications, such as pelvic inflammatory disease, ectopic pregnancy and infertility (39–43). Similar levels of *C. trachomatis* EBs and intracellular inclusions were detected in the lower and upper genital tract tissues of the infection and the re-infected group. This indicates that *C. trachomatis* L2c could colonize the urogenital tract following re-infection as efficiently as following primary infection. Thus, the primary infection failed to induce protective immune responses against re-infection. Several epidemiologic studies of humans indicate that protective immunity to re-infection, albeit only partial, develops following prior genital infection with *C. trachomatis*, including LGV strains (44–49). These findings are consistent with those from murine and non-human primate model experiments (50–52).

In our study, the infection was confined to the urogenital tract with infection of the tissues from the vagina to the ovaries and including the urethra. There was no evidence of chlamydial bacteria in the caecum, spleen, liver, and pelvic lymph node samples of the infected animals. This is in accordance with the symptoms observed in the patient from which L2c was isolated (25). The patient suffered from severe localized mucosal disease, namely hemorrhagic proctitis, and did not show an inguinal syndrome, indicating that the bacteria had not spread to the inguinal lymph nodes. This lack of dissemination to regional lymph nodes was likely due to the presence of a functional cytotoxin, which probably enables innate immune evasion (53, 54). The toxin gene was presumably acquired from a D strain, since all other LGV strains to date lack this gene (25).

Infection of the female porcine genital tract with *C. trachomatis* serovar L2c resulted only in minor microscopic lesions, in particular interepithelial inflammatory cells and epithelial vacuolation. This is in contrast with a previous study describing genital tract infection of female pigs with two *C. trachomatis* serovar E strains (31). However, samples in the present study and the study by Vanrompay et al., (31) were not taken at the same time point post infection and the infection schedule was different. Remarkably, histopathological changes were more prominent in the genital tissues of the infected group than the re-infected group, which is opposed to the gross pathology noticed in both groups. Immunohistochemistry could provide more detailed information on the cells involved in the microscopic lesions and therefore will be performed during future *C. trachomatis* experiments in pigs.

All infected animals vaginally excreted *C. trachomatis* from three days post infection onwards. Prior infection had no influence on vaginal chlamydial shedding, since similar excretion levels were observed after primary infection and re-infection. Vaginal excretion was consistently high until 49 days post primary infection and was only slightly decreased at day 56, indicating that the pigs had not yet resolved the chlamydial infection after 8 weeks. In a future experiment, some animals will be kept longer, monitoring bacterial excretion twice a week to find out if the infection will be cleared. In humans, *C. trachomatis* infection can last several months before spontaneous clearance (55–57). In some patients infected with LGV serovars, the bacteria could still be isolated many years after the primary infection (58, 59).

Anti-chlamydial IgM, IgG and IgA antibodies were elicited in sera and vaginal secretions following primary infection with *C. trachomatis* L2c. Re-infection clearly resulted in a secondary systemic and mucosal antibody response, with significantly lower IgM titers and significantly higher IgG and IgA titers than following primary infection. However, the induced antibody response was not able to even partially protect the pigs against re-infection. The importance of antibodies in immunity to *C. trachomatis* is still disputed. In women, the amount of IgA in cervical secretions was found to correlate inversely with the amount of *C. trachomatis* recovered from the cervix (60). However, high titers of *C. trachomatis*-specific serum IgG antibodies were not associated with resolution of infection in women, but with increased severity of post-infection complications (61). In mice, antibodies are not needed to resolve a primary genital *C. muridarum* infection (62), but contribute to protection against re-infection (63). In the guinea pig model, antibodies play an important role in immunity to both primary infection and re-infection with *Chlamydia caviae* (64, 65). Interestingly, it was shown that *C. trachomatis* strains of the LGV biovar are more resistant to neutralization by immune sera than strains of the trachoma biovar (66), which may explain the lack of protection on re-infection in our study.

The proliferative responses of PBMC and MC to *C. trachomatis* strain L2c were never statistically different among groups, suggesting that *C. trachomatis*-specific lymphocytes were not generated following infection or re-infection. However, anti-chlamydial antibodies were detected, which implies that *C. trachomatis*-specific B lymphocytes must have been created at a certain point. Simultaneously with the proliferation assays, the immune cell subpopulations within PBMC and lymphoid tissue MC were analyzed. The observed differences in percentages of B and T lymphocytes among groups are considered irrelevant, since no *C. trachomatis*-specific lymphocytes appeared to be present at the examined time points. Furthermore, the differences in percentages of monocytes, NK cells and pDC between groups were sometimes inconsistent. Primary infection of the re-infected group resulted in a significant increase of NK-cells in the blood, whereas this was not the case following primary infection of the infected group. However, both the spleen and the pelvic lymph nodes of the re-infected animals contained significantly higher percentages of NK cells three weeks post re-infection. NK cells are an important component of the innate immune response, which can help control chlamydial infections by secreting IFN-γ (67). Moreover, it was shown that human NK cells are able to lyse epithelial cells infected with *C. trachomatis* (68). Nevertheless, an effective protective immune response to genital *C. trachomatis* infections is highly dependent on specific CD4^+^ T-helper Type 1 cells (69–71), but also on IFN-γ (72, 73). The apparent lack of *C. trachomatis*-specific T cells following infection or re-infection in the present study can explain why the pigs were not even partially protected against re-infection. However, the immune responses detected in the blood, the spleen, and cervical and pelvic lymph nodes do not necessarily mirror the immune responses elicited at the local site of infection. It would have been interesting to obtain immunohistochemical data on genital tract tissues as this could provide more information about the local immune response following infection or re-infection. Immunohistochemistry on genital tract tissues was performed, but without success. The available porcine leukocyte markers could not be used on formalin fixed samples notwithstanding the use of the eBioscience™ IHC antigen retrieval system (unpublished data). In the future, samples for immunohistochemistry will be collected in an in house-made zinc phosphate fixative as it allows optimal antigen retrieval and visualization of porcine immune cell populations in an “*in situ*” histological setting (unpublished data).

Thus, *C. trachomatis* serovar L2c infection of pigs failed to induce protective immune responses against reinfection. Similar results were observed in mice after primary and secondary *C. muridarum* or *C. trachomatis* serovar E infections (74–76). Also, regarding pathology in women, increases in complications associated with chlamydial infections, occurred in recurrent infections (77). Both the pathogen and the host contribute to the outcome of chlamydial infections. Recently, genome-wide profiling of humoral immunity and pathogen genes under selection identified immune evasion tactics of *C. trachomatis* during ocular infection in children in The Gambia (78). Children who were susceptible to frequent and/or prolonged ocular *C. trachomatis* infection had a less focused antibody response and data suggested a strategy in which *C. trachomatis* presents a large panel of irrelevant antigens to the human immune system to block or misdirect protective responses. The latter might also have occurred during the present study as serum and mucosal antibody titers against L2c elementary bodies where not particularly high. In addition, several research groups provided insight in human (76) and murine (79–82) small non-coding RNA (miRNA) expression profiles during genital and ocular chlamydial infection and reinfection and found chlamydia virulence to be associated with the miRNA expression profile of the host. miRNAs regulate gene expression as they target mRNAs for degradation, cleavage, translational repression or occasional enhancement. Specific miRNAs were involved in the primary infection and reinfection resulting in a different pathogenesis and outcome in pathology, being more severe in reinfected mice than in primary infected animals. It might be interesting to examine this in the pig model.

In conclusion, the pathogenesis of an L2c infection in the porcine model is rather similar to the pathogenesis of L2c and other *C. trachomatis* strain infections in humans. Intravaginal inoculation of pigs with *C. trachomatis* L2c resulted in an infection that was confined to the urogenital tract, where inflammation and pathology were caused. Re-infection resulted in more severe gross pathology than primary infection but chlamydial colonization of the urogenital tract was similar for both the primary infected and re-infected groups. This indicates that primary infection failed to induce protective immune responses against re-infection. Indeed, no *C. trachomatis*-specific lymphocytes could be detected in the blood and the lymphoid tissues following infection or re-infection. However, the cellular immune responses need to be further investigated by performing immunohistochemical analysis of the infected urogenital tissues. Hereto, a zinc phosphate fixative will be used instead of formalin. Nevertheless, anti-chlamydial antibodies were elicited in sera and vaginal secretions after primary infection, and re-infection clearly resulted in a secondary systemic and mucosal antibody response. Apparently, the induced antibody response could not even partially protect the pigs against re-infection. Our study supports the notion that the porcine model is an alternative large animal model for evaluating the immunopathogenesis and evolution of *C. trachomatis* infections that will provide data for advancing therapeutic and vaccine development for humans. Our findings imply that the pig may function as either an intermediate animal model between mouse and non-human primates or as a substitute for the latter during the development of therapeutics and vaccines for use in human clinical trials.

## Data Availability Statement

All datasets presented in this study are included in the article/**Supplementary Material**.

## Ethics Statement

The animal study was reviewed and approved by Ethische Commissie van de Faculteit Diergeneeskunde en Bio-ingenieurswetenschappen van de Universiteit Gent.

## Author Contributions

DV, EC, and DD contributed to conception and design of the study. EDC wrote the first draft of the manuscript and conducted the experiment together with MG and KS. BD assisted during flow cytometry. KC performed histology. WB performed immunohistochemistry. CK prepared the final version of the manuscript. All authors contributed to the article and approved the submitted version.

## Funding

The research was supported by the Special Research Fund of Ghent University (BOF).

## Conflict of Interest

The authors declare that the research was conducted in the absence of any commercial or financial relationships that could be construed as a potential conflict of interest.
