# Supplementary material for: Chlamydia trachomatis L2c Infection in a Porcine Model Produced Urogenital Pathology and Failed to Induce Protective Immune Responses Against Re-Infection
Source: Front Immunol. 2020 Oct 26;11:555305. doi: 10.3389/fimmu.2020.555305 (PMC7649141; doi:10.3389/fimmu.2020.555305)
Supplement: Supplementary file 1 [file Table_1.docx]

Supplementary Material

**Supplementary Table 1. Overview of the monoclonal antibodies used in flow cytometry.**

| Specificity | Clone | Isotype | Reference |
| --- | --- | --- | --- |
| CD3 | PTT3 | IgG1 | (74) |
| CD4 | 74-12-4 | IgG2b | (75) |
| CD8α | 11/295/33 | IgG2a | (76) |
| CD8β | PG164A | IgG2a | (77) |
| CD21 | BB6-11C9 | IgG1 | (77) |
| IgM | 28.4.1 | IgG1 | (37) |
| MHCII | MSA3 | IgG2a | (78) |
| SWC3a | 74-22-15 | IgG1 | (75) |
| TCR1-N4 (δ chain) | PGBL22A | IgG1 | (79) |

**Supplementary Table 2. Gross lesions detected at euthanasia in the infected and the re-infected group^#^.**

|  |  | - Median score* (range, % of positive animals) in: | |
| --- | --- | --- | --- |
| - Tissue | - Macroscopic - lesion | - Infected group | - Re-infected group |
| Vagina | Congestion | 0 (0-2, 20) | 0 (0-2, 40) |
|  | Serous exudate | 0 (0-3, 40) | 0 (0-3, 40) |
| Cervix | Congestion | 0 (0-2, 20) | 0 (0-0, 0) |
|  | Serous exudate | 0 (0-3, 40) | 0 (0-3, 40) |
| Uterus | Congestion | 0 (0-1, 20)*^c^* | 1 (1-3, 100)*^b,c^* |
|  | Hypertrophy | 0 (0-2, 20) | 0 (0-1, 20) |
|  | Serous exudate | 0 (0-3, 40) | 3 (0-3, 60)*^b^* |
| Uterine tubes | Congestion | 0 (0-1, 40)*^c^* | 1 (1-3, 100)*^b,c^* |
|  | Serous exudate | 0 (0-3, 40) | 0 (0-3, 40) |
| Oviducts | Serous exudate | 0 (0-2, 20) | 0 (0-2, 40) |
|  | Dilated | 0 (0-2, 20) | 2 (0-2, 80)*^b^* |
| *Lig. latum uteri* | Congestion | 1 (0-2, 60) | 2 (0-3, 80)*^b^* |
| Mesovarium | Congestion | 2 (0-3, 60) | 2 (0-3, 80)*^b^* |
| Urethra | Congestion | 0 (0-3, 40) | 3 (1-3, 100)*^b^* |
| Spleen | Congestion | 0 (0-2, 20) | 0 (0-3, 40) |
|  | Enlargement | 0 (0-0, 0) | 0 (0-3, 20) |
|  | red pulp expansion | 0 (0-0, 0) | 0 (0-2, 20) |
|  | white pulp expansion | 0 (0-3, 40) | 0 (0-2, 40) |
| Liver | Congestion | 0 (0-0, 0) | 0 (0-2, 40) |
| Pelvic lymph nodes | Congestion | 2 (0-3, 80)*^a^* | 3 (0-3, 80)*^b^* |
|  | Enlargement | 2 (2-3, 100)*^a^* | 3 (2-3, 100)*^b^* |

^#^ The median score (range) in the control group was 0 (0-0) for all tissues.

* Lesions were scored as none (0), slight (1), moderate (2) or severe (3).

*^a^* P < 0.05 for a comparison of the control group and the infected group.

*^b^* P < 0.05 for a comparison of the control group and the re-infected group.

*^c^* P < 0.05 for a comparison of the infected group and the re-infected group.

**Supplementary Table 3.** **Histopathological findings in the urogenital tract at euthanasia*.**

|  |  | Median score (range) in: | | |
| --- | --- | --- | --- | --- |
| Tissue | Histopathological findings^#^ | Control  group | Infected  group | Re-infected  group |
| Vagina | Interepithelial inflammatory cells | 0 (0-0) | 0 (0-1) | 0 (0-0) |
|  | Infiltration of pmc in the lamina propria | 0 (0-0) | 0 (0-1) | 0 (0-0) |
|  | Infiltration of mnc in the lamina propria | 1 (1-2) | 0 (0-2) | 0 (0-1) |
| Cervix | Interepithelial inflammatory cells | 0 (0-1)**^a^** | 2 (1-4)**^a,c^** | 0 (0-2)**^c^** |
|  | Degeneration and/or apoptosis of epithelial cells | 0 (0-0)**^a^** | 1 (0-2)**^a^** | 0 (0-2) |
|  | Infiltration of pmc in the lamina propria | 0 (0-0) | 0 (0-1) | 0 (0-0) |
|  | Infiltration of mnc in the lamina propria | 0 (0-2) | 2 (0-3) | 1 (0-3) |
|  | Oedema lamina propria | 0 (0-0) | 0 (0-0) | 0 (0-1) |
| Corpus | Interepithelial inflammatory cells | 1 (0-2) | 1 (0-2) | 0 (0-2) |
| uteri | Degeneration and/or apoptosis of epithelial cells | 0 (0-1)**^a^** | 3 (0-3)**^a^** | 1 (0-2) |
|  | Infiltration of mnc in the lamina propria | 1 (1-2) | 2 (1-2) | 1 (1-2) |
|  | Oedema lamina propria | 0 (0-1) | 0 (0-1) | 2 (0-2) |
| Uterine | Interepithelial inflammatory cells | 1 (1-2) | 2 (1-3) | 1 (1-1) |
| Horn R | Degeneration and/or apoptosis of epithelial cells | 0 (0-0)**^a^** | 2 (1-3)**^a^** | 1 (0-2) |
|  | Infiltration of mnc in the lamina propria | 2 (1-2) | 2 (1-3) | 1 (1-2) |
|  | Oedema lamina propria | 1 (0-1) | 1 (0-1) | 1 (0-3) |
| Uterine | Exfoliation | 0 (0-1) | 0 (0-0) | 0 (0-0) |
| Horn L | Interepithelial inflammatory cells | 1 (0-2) | 2 (1-3) | 1 (1-2) |
|  | Degeneration and/or apoptosis of epithelial cells | 0 (0-1)**^a,b^** | 2 (1-4)**^a^** | 1 (1-2)**^b^** |
|  | Infiltration of pmc in the lamina propria | 0 (0-0) | 0 (0-0) | 0 (0-1) |
|  | Infiltration of mnc in the lamina propria | 2 (1-3) | 2 (1-2) | 1 (0-2) |
|  | Oedema lamina propria | 1 (0-1) | 1 (0-1) | 1 (0-3) |
| Oviduct | Intraluminal proteinaceous fluid | 0 of 5 | 2 of 5 | 0 of 5 |
| R | Exfoliation | 0 (0-0) | 1 (0-3) | 0 (0-0) |
|  | Infiltration of mnc in the lamina propria | 1 (0-2) | 0 (0-1) | 0 (0-2) |
| Oviduct | Intraluminal proteinaceous fluid | 1 of 5 | 0 of 5 | 1 of 5 |
| L | Infiltration of mnc in the lamina propria | 1 (0-3) | 1 (0-2) | 2 (0-2) |
| Urethra | Degeneration and/or apoptosis of epithelial cells | 0 (0-0)**^b^** | 0 (0-2) | 2,5 (1-4)**^b^** |
|  | Infiltration of mnc in the lamina propria | 0 (0-1) | 0 (0-3) | 0 (0-0) |

* The median score (range) for the histopathological parameters which are not shown in the table, were 0 (0-0) for the three groups. ^#^ Exfoliation: superficial layer of exfoliated (epithelial) cells and/or inflammatory cells; pmc: polymorphonuclear inflammatory cells; mnc: mononuclear inflammatory cells. **^a^** P < 0.05 for a comparison of the control group and the infected group. **^b^** P < 0.05 for a comparison of the control group and the re-infected group. **^c^** P < 0.05 for a comparison of the infected group and the re-infected group.

**Supplementary Table 4. Vaginal *C. trachomatis* shedding from 0 to 77 days post infection (dpi) in the infected and the re-infected group^#^.**

|  | Median score* (range) in: | |
| --- | --- | --- |
| dpi | Infected group | Re-infected group |
| 0 | 0 (0-0) | 0 (0-0) |
| 3 | 0 (0-0)*^c^* | 3 (1-3)*^b,c^* |
| 7 | 0 (0-0)*^c^* | 3 (3-4)*^b,c^* |
| 10 | 0 (0-0)*^c^* | 3 (1-3)*^b,c^* |
| 14 | 0 (0-0)*^c^* | 3 (3-3)*^b,c^* |
| 21 | 0 (0-0)*^c^* | 3 (3-4)*^b,c^* |
| 28 | 0 (0-0)*^c^* | 3 (3-4)*^b,c^* |
| 35 | 0 (0-0)*^c^* | 3 (3-3)*^b,c^* |
| 42 | 0 (0-0)*^c^* | 3 (2-3)*^b,c^* |
| 49 | 0 (0-0)*^c^* | 3 (1-3)*^b,c^* |
| 56 | 0 (0-0)*^c^* | 1 (0-3)*^b,c^* |
| 59 | 3 (3-3)*^a^* | 1 (1-3)*^b^* |
| 63 | 3 (1-3)*^a^* | 3 (1-3)*^b^* |
| 66 | 3 (3-3)*^a^* | 3 (1-3)*^b^* |
| 70 | 3 (3-3)*^a^* | 3 (3-3)*^b^* |
| 77 | 3 (3-3)*^a^* | 3 (1-3)*^b^* |

^#^ The median score (range) in the control group was 0 (0-0) for all time points.

* *C. trachomatis* positive cells were counted in five randomly selected microscopic fields. Score 0: no *C. trachomatis* positive cells; Score 1: 1-5 EBs and no inclusions; Score 2: 6-10 EBs and no inclusions; Score 3: >10 EBs and 1 inclusion-positive cell; Score 4: 1-5 inclusion-positive cells; Score 5: 6-10 inclusion-positive cells; Score 6: >10 inclusion-positive cells.

*^a^* P < 0.05 for a comparison of the control group and the infected group.

*^b^* P < 0.05 for a comparison of the control group and the re-infected group.

*^c^* P < 0.05 for a comparison of the infected group and the re-infected group.
